# Driving factors in treatment decision-making of patients seeking medical assistance for infertility: a systematic review

**DOI:** 10.1093/humupd/dmae001

**Published:** 2024-02-01

**Authors:** Felicia von Estorff, Monique H Mochtar, Vicky Lehmann, Madelon van Wely

**Affiliations:** Department of Obstetrics and Gynaecology, Centre for Reproductive Medicine, Amsterdam UMC, University of Amsterdam, Amsterdam, The Netherlands; Department of Obstetrics and Gynaecology, Centre for Reproductive Medicine, Amsterdam UMC, University of Amsterdam, Amsterdam, The Netherlands; Amsterdam Reproduction and Development Research Institute, Amsterdam, The Netherlands; Amsterdam Reproduction and Development Research Institute, Amsterdam, The Netherlands; Department of Medical Psychology, Amsterdam UMC, University of Amsterdam, Amsterdam, The Netherlands; Department of Obstetrics and Gynaecology, Centre for Reproductive Medicine, Amsterdam UMC, University of Amsterdam, Amsterdam, The Netherlands; Amsterdam Reproduction and Development Research Institute, Amsterdam, The Netherlands

**Keywords:** infertility, patient preference, child-wish, assisted reproduction, acceptability of healthcare, ART

## Abstract

**BACKGROUND:**

ART differs in effectiveness, side-effects, administration, and costs. To improve the decision-making process, we need to understand what factors patients consider to be most important.

**OBJECTIVE AND RATIONALE:**

We conducted this systematic review to assess which aspects of ART treatment (effectiveness, safety, burden, costs, patient-centeredness, and genetic parenthood) are most important in the decision-making of patients with an unfulfilled wish to have a child.

**SEARCH METHODS:**

We searched studies indexed in Embase, PubMed, PsycINFO, and CINAHL prior to November 2023. Discrete choice experiments (DCEs), surveys, interviews, and conjoint analyses (CAs) about ART were included. Studies were included if they described two or more of the following attributes: effectiveness, safety, burden, costs, patient-centeredness, and genetic parenthood.

Participants were men and women with an unfulfilled wish to have a child. From each DCE/CA study, we extracted the beta-coefficients and calculated the relative importance of treatment attributes or, in case of survey studies, extracted results. We assessed the risk of bias using the rating developed by the Grading of Recommendations Assessment, Development and Evaluation working group. Attributes were classified into effectiveness, safety, burden, costs, patient-centeredness, genetic parenthood, and others.

**OUTCOMES:**

The search identified 938 studies of which 20 were included: 13 DCEs, three survey studies, three interview studies, and one conjoint analysis, with a total of 12 452 patients. Per study, 47–100% of the participants were women. Studies were assessed as having moderate to high risk of bias (critical: six studies, serious: four studies, moderate: nine studies, low: one study). The main limitation was the heterogeneity in the questionnaires and methodology utilized. Studies varied in the number and types of assessed attributes. Patients’ treatment decision-making was mostly driven by effectiveness, followed by safety, burden, costs, and patient-centeredness. Effectiveness was rated as the first or second most important factor in 10 of the 12 DCE studies (83%) and the relative importance of effectiveness varied between 17% and 63%, with a median of 34% (moderate certainty of evidence). Of eight studies evaluating safety, five studies valued safety as the first or second most important factor (63%), and the relative importance ranged from 8% to 35% (median 23%) (moderate certainty of evidence). Cost was rated as first or second most important in five of 10 studies, and the importance relative to the other attributes varied between 5% and 47% (median 23%) (moderate certainty of evidence). Burden was rated as first or second by three of 10 studies (30%) and the relative importance varied between 1% and 43% (median 13%) (low certainty of evidence). Patient-centeredness was second most important in one of five studies (20%) and had a relative importance between 7% and 24% (median 14%) (low certainty of evidence). Results suggest that patients are prepared to trade-off some effectiveness for more safety, or less burden and patient-centeredness. When safety was evaluated, the safety of the child was considered more important than the mother’s safety. Greater burden (cycle cancellations, number of injections, number of hospital visits, time) was more likely to be accepted by patients if they gained effectiveness, safety, or lower costs. Concerning patient-centeredness, information provision and physician attitude were considered most important, followed by involvement in decision-making, and treatment continuity by the same medical professional. Non-genetic parenthood did not have a clear impact on decision-making.

**WIDER IMPLICATIONS:**

The findings of this review can be used in future preference studies and can help healthcare professionals in guiding patients’ decision-making and enable a more patient-centered approach.

## Graphical Abstract

**Figure:**
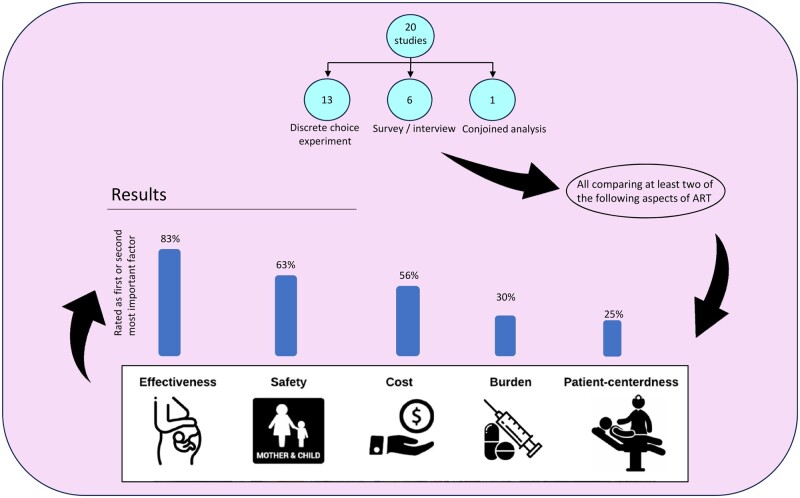
**In patients seeking medical assistance for infertility, the most important attribute driving patients’ decision-making was effectiveness of treatment followed by safety and costs; burden and patient-centeredness were considered less important**.

## Introduction

Patients seeking medical assistance to fulfill their wish to have a child can utilize a wide range of ART. Technologies such as IVF, ICSI, frozen embryo transfer, or preimplantation genetic screening may result in reasonable chances for a pregnancy but differ in treatment duration, side-effects, and safety for mother and child—aspects that affect patient preferences. The availability of multiple reasonable treatment options makes treatment decisions highly preference-sensitive (van der Horst *et al.*, 2023). Besides differences in treatment-related aspects, variability in costs may also affect decision-making, although in some countries fertility treatments are covered by health insurance with few out-of-pocket costs remaining for patients. Furthermore, using a patient-centered approach, for instance by providing more information and helping in shared decision-making, may affect how treatment options are perceived (Malin *et al.*, 2001; Twisk *et al.*, 2007). Technologies are rapidly advancing and new treatment options make decision-making even more complex, highlighting the need to know what factors are important to patients.

To elicit patient preferences in a quantitative way, discrete choice experiments (DCEs) and conjoint analyses (CAs) have been established as a way to quantify preferences. These are survey-based research methods used to understand how people make decisions when presented with multiple options. In a DCE, participants are typically presented with a series of hypothetical scenarios where they must choose between two scenarios. For example, a DCE might ask participants to choose between different treatment options based on attributes such as pregnancy chance, side-effects, number of hospital visits, and supervision by single or multiple providers. To ensure that the attributes are relevant, they are usually defined by expert focus groups and literature reviews (Botha *et al.*, 2018). By analyzing participants’ choices, researchers can determine which attributes are considered most important when making decisions. Besides allowing us to determine trade-offs that patients are prepared to make, DCEs also allow us to objectively assess the relative importance patients place on the attributes of the scenarios (Reed Johnson *et al.*, 2013). DCEs are commonly used in marketing research, health economics, and environmental economics to understand consumer preferences and predict behavior.

In a CA, treatment attributes and levels are used as well. While in a DCE patients choose between different choice sets, in a CA, patients are asked to rank hypothetical treatment scenarios from best to worst (Bridges *et al.*, 2011; Louviere *et al.*, 2010) in order to draw conclusions about ‘willingness to pay’ or trade-offs. Besides DCEs and conjoined designs, surveys and interviews are often used to evaluate patient preferences. Such studies have the advantage of being more flexible and with the potential to capture new ideas.

All these preference studies help to better understand how men and women value different aspects of their treatment. This allows clinicians to better respond to patient expectations, to enable patients’ decision-making, and to improve decision aids (Klitzman, 2018).

With this systematic review, we aimed to study what attributes patients with an unfulfilled wish to have a child consider to be most important when making treatment decisions about ART.

## Methods

This systematic review followed the reporting guidelines of the PRISMA 2020 Statement (Page *et al*., 2021). The study protocol was registered in Prospero: CRD42022302551.

### Eligibility criteria

Studies were considered eligible if preferences related to ART were evaluated in men and/or women with an unfulfilled wish to have a child. As technologies and preferences may change over time, only articles published since January 2000 were included. These had to be designed as a DCE, conjoint experiment, survey, or structured interview using quantitative techniques (i.e. varying the probability of pregnancy until preferences regarding ART techniques changed).

We excluded studies regarding fertility preservation in cancer, gonadotrophin application, and birth outcomes only. We also excluded studies that did not describe attributes related to at least two of the following: costs, safety, burden, effectiveness, genetic parenthood, and patient-centeredness.

### Literature search

To identify eligible studies, we searched the databases Embase, PsycINFO, PubMed, and CINAHL from 1 January 2000 to 20 November 2023, without any language restriction. Additionally, we checked the reference lists of included studies. Our search strategy can be found in Supplementary Data File S1.

### Study selection and critical appraisal

Two authors (M.v.W. and F.v.E.) independently screened the title and abstract of all identified publications. After initial screening, the full-text articles of studies identified as relevant were further evaluated by the same two authors. Disagreements were resolved by discussion until consensus was reached.

Two authors (M.v.W. and F.v.E.) assessed the risk of bias of each included study independently. Disagreements were resolved by consensus. Risk of bias assessments for preference studies were based on a rating developed by the Grading of Recommendations Assessment, Development and Evaluation (GRADE) working group (Zhang *et al.*, 2019). The following criteria were assessed: participant selection, completeness of data, measurement instruments, and data analysis. These were rated as low, moderate, serious, or critical. Subsequently, we graded the evidence using the approach of the same GRADE working group (Zhang *et al.*, 2019).

### Data extraction and outcomes

Data were extracted from the studies, including:

*Study characteristics*: author, year of publication, country, preference elicitation method, response rate, type of assessed attributes, and number of attributes.

*Patient characteristics*: number of patients, gender, duration of child wish, education, and income.

*Outcomes*: study attributes were recorded. We aimed to combine the attributes, if possible, into the following attribute groups: effectiveness (chance to conceive, live birth rate, time to successful pregnancy), safety (ovarian hyperstimulation syndrome (OHSS), side effects, neonatal or maternal complications), burden (cycle cancellations, number of injections, hospital visits), patient-centeredness (patient involvement, continuity of physicians, information provision), cost, genetic parenthood, and others (e.g. moral acceptability, availability of experimental treatments).

For the DCE and CA designed studies, we present descriptive data of the relative importance of attributes and visually present the attributes that were chosen as most important or second most important. We also describe the trade-offs that patients were willing to make.

For the survey and interview studies, data representing the importance of examined attributes are described.

### Data analysis

For DCE and CA designs, the effects of studied attributes were measured as beta-coefficients, and as relative measures of willingness to trade between attributes. We calculated the relative importance of attributes by first standardizing the beta-coefficients per study by taking the coefficient of the highest levels per attribute divided by the standard error. These standardized coefficients were subsequently translated to a scale ranging from 0 to 1, by dividing by the sum of all standardized coefficients. In case of multiple attributes per attribute group, we took the largest effect (largest beta) to calculate the relative importance.

From survey and interview studies, the effect of attributes was presented descriptively. This allowed us to extract the relative value of an attribute (versus the other studied attributes) as more important, equally important or less important.

As each study included a different combination and number of attributes, it was not possible to combine these estimates in a meta-analysis.

## Results

### Search results

**Figure 1.**
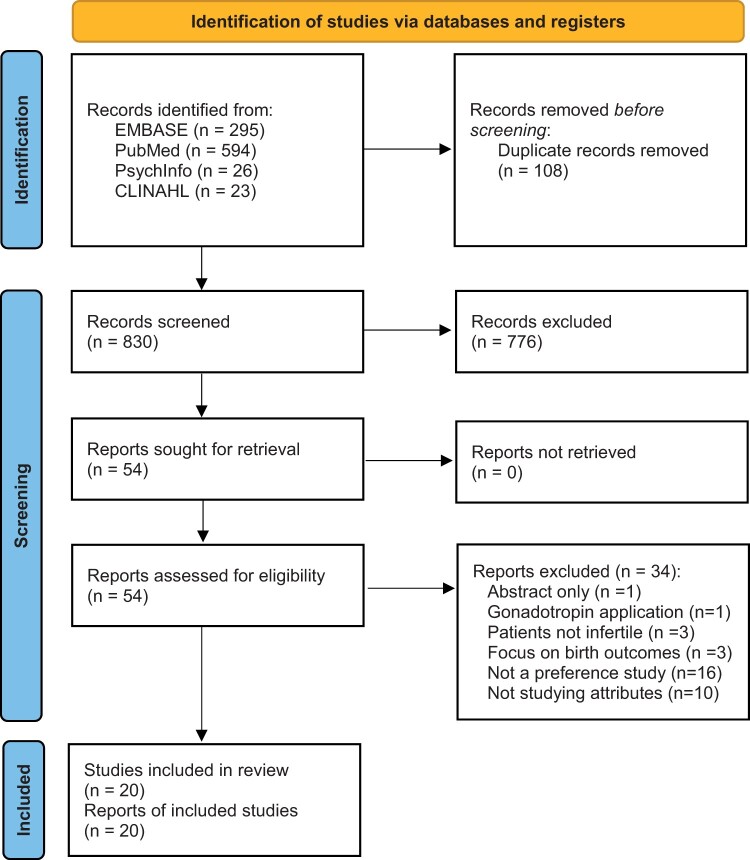
**PRISMA flow diagram of the literature search for studies of factors driving treatment decision-making by patients seeking medical assistance for infertility**.

The electronic search generated 938 studies. After removing duplicates, the title and abstract of 830 studies were screened of which 54 were included for full-text review. Of these, 20 studies met inclusion criteria (Fig. 1).

### Study characteristics

The characteristics of included studies are described in Table 1. Twelve studies had a DCE design (Musters *et al.*, 2011; van Empel *et al.*, 2011; Huppelschoten *et al.*, 2014; van den Wijngaard *et al.*, 2014, 2015; Weiss *et al.*, 2017; Hendriks *et al.*, 2019; Braam *et al.*, 2020; Abdulrahim *et al.*, 2021; Cornelisse *et al.*, 2022; Skedgel *et al.*, 2022; Keller *et al.*, 2023), one study included both a DCE and a survey (Hendriks *et al.*, 2016), one was designed as CAs study (Palumbo *et al.*, 2011), and six were survey and/or interview studies (Bayram *et al.*, 2005; Steures *et al.*, 2005; van Weert *et al.*, 2007; Sills *et al.*, 2012; Duthie *et al.*, 2017; Stormlund *et al.*, 2019).

**Table 1.** Characteristics of studies included in a systematic review of factors driving treatment decision-making by patients seeking medical assistance for infertility.

Author, year	Design	Setting	Number of patients	Percentage of women	Response rate	Mean age (years)	Duration childwish (months)	Income	Education	Types of attributes	Number of attributes
Braam, 2020	DCE	Netherlands	91	100%	79%	30.2	8	Low (3.3%), moderate (38,5%), high (47.3%), NR (11.0%)	Low (11%), moderate (44%), high (45.1%)	Three burden, safety, costs	5
Keller, 2023	DCE	Australia	376	100%	Unknown	Unclear	Unknown	<$25k (6%), $25–$50k (11%), $50–$100k (36%), $100–$150k (24%), >$150k (13%), NR(9%)	Not finished high school (8.2%), high school (11.4%), Certificate/trade/TAFE (36.2%), University (44.2%)	Pregnancy rate, burden, three patient-centeredness, availability of experimental treatment, cost	7
Hendriks, 2019	DCE	Netherlands, Belgium	173	54%	32%	37	52.8	Higher than modal 127 (73%), equal or less than modal (13%), NR (13%)	University degree (62%), no university degree (38%)	Two safety, costs, pregnancy rate, genetic parenthood, curing infertility	6
van Empel, 2011	DCE	Netherlands, Belgium	925	52%	67%	34	33.6	Unknown	Low-median (48%), high (52%)	Pregnancy rate, three patient-centeredness, burden	5
van den Wijngaard, 2015	DCE	Netherlands	93	100%	77%	33	Unknown	Low (12%), moderate (30%), high (55%), NR (3%)	Not finished (2%), secondary (7%), vocational (36%), college/university (53%), NR 2 (2%)	Two burden, safety	3
Weiss, 2017	DCE	Netherlands	145	100%	Unknown	30	12.6	Below average (3.4%), average (22.1%), above average (69.7%), NR (4.9%)	Primary (0%), secondary (20%), tertiary (80%)	Three burden, cost, pregnancy rate, place of fertilization	5
Huppelschoten, 2014	DCE	Netherlands	550	51.20%	55.20%	35	28	At or below modal income (21.5%), ≥1.5× modal income (78.5%)	Low–middle (51.2%), high (48.8%)	Three patients centered, pregnancy rate, costs	5
Musters, 2011	DCE	Netherlands	206	100%	95%	Unclear: 52% ≥35 years old 48% <35	37.7	Very low (13%), low (29%), moderate (23%), high (28%), NR (8%)	Low (9%), moderate (31%), high (59%), NR (1%)	Pregnancy rate, burden, costs	3
Abdulrahim, 2021	DCE	UK	208	50.5%	58.80%	37	38.1	Up to £20 000 (7.2%), £20 000–40 000 (28.4%), £40 000–over 50 000 (61.5%), NR (2.9%)	Unknown	Pregnancy rate, safety, cost, chance of miscarriage	4
van den Wijngaard, 2014	DCE	Netherlands	172	100%	Unknown	Unknown	Unknown	Unknown	Unknown	Pregnancy rate, two burden, safety	4
Palumbo, 2011	CA	Spain	160	100%	Unclear	35.8	Unknown	Net income <1502 (50.0%), income of >1502 (40.8%), income between 1804 and 2404 (9.2%)	No school (0.6%), primary (13.8%), secondary (16.3%), college (14,4%), university (55.0%)	Costs, patient-centeredness, safety, pregnancy rate, burden	5
Sills, 2012	Survey	USA, CA	71	Unclear	100%	34	28.8	Unknown	College (33.8%), no college (66.2%)	Costs, burden	2
Duthie, 2017	Survey	USA, WI	118	50%	19%	Unclear	Unknown	<$40k (28.8%), $40–$60k (27%), $60–$80k (20%), ≥$80k (20.3%), NR (3.4%)	No degree (27.1%), college degree (39.8%), advanced degree (30.5%), NR (2.5%)	Costs, two genetic parenthood, burden, two pregnancy rate	6
Skedgel, 2022	DCE	USA, UK, Denmark, Nordic (Norway, Sweden, Finland), Spain, China	7565	China—57%, Nordic countries—58%, Spain-52%, UK—59%, USA—59%	Unknown	Unclear: between 31 and 45 years of age.	Unclear: majority over 12 months	China 52% and Nordic 35%, (£30–45k). Spain (51%) and UK (33%) (£15–30k). USA (24%) (£45k–60k)	Unknown	Pregnancy rate, safety, two burden, patient centeredness, and cost	6
Hendriks, 2016	Survey	Netherlands	988	Unclear	54%	35.4	62.6	Unknown	High (48.5%)	Burden, cost, curing infertility, conception at home, naturalness, moral acceptability, technological sophistication, safety for child, and pregnancy rate	9
Stormlund, 2019	Survey	Denmark	165	62.00%	77.1%	34.3	28.8	Social class only women: 38% upper, 41% middle and 12% low men: 44% upper, 40% middle, 5% low	Unknown	Safety (OHSS), safety (of child), pregnancy rate, burden (time), and a natural process	5
Cornelisse, 2022	DCE	Netherlands	164	100%	36.80%	34	Unknown	Unknown	High (75%), medium and low (25%)	Pregnancy rate, two burden (time to pregnancy and quality of life), and number of embryos	4
van Weert, 2007	Interview	Netherlands	146	50%	Unknown	34.4	44.4	Unknown	Unknown	Pregnancy rate, safety (OHSS), and risk of multiple pregnancies	3
Steures, 2005	Interview	Netherlands	80	50%	Unknown	33	22	Unknown	Unknown	Pregnancy rate, safety (OHSS), and risk of multiple pregnancies	3
Bayram, 2005	Interview	Netherlands	64	100%	80%	Unknown	Unknown	Unknown	Unknown	Pregnancy rate, safety, and burden	3

OHSS: ovarian hyperstimulation syndrome; DCE: discrete choice experiment; CA: conjoined analysis; NR: not reported.

Of the 20 studies, 11 had been conducted in the Netherlands (Bayram *et al.*, 2005; Steures *et al.*, 2005; van Weert *et al.*, 2007; Musters *et al.*, 2011; van Empel *et al.*, 2011; Huppelschoten *et al.*, 2014; van den Wijngaard *et al.*, 2014, 2015; Weiss *et al.*, 2017; Braam *et al.*, 2020; Cornelisse *et al.*, 2022), two studies were conducted in both the Netherlands and Belgium (Hendriks *et al.*, 2016, 2019), two studies were carried out in the USA (Sills *et al.*, 2012; Duthie *et al.*, 2017), one in Australia (Keller *et al.*, 2023), one in the UK (Abdulrahim *et al.*, 2021), one in Denmark (Stormlund *et al.*, 2019), and one in Spain (Palumbo *et al.*, 2011). The largest study included multiple countries: the USA, the UK, Denmark, Norway, Sweden, Finland, Spain, and China (Skedgel *et al.*, 2022).

The number of attributes ranged between two and nine and the average number of attributes used per study was five.

### Patient characteristics

In total, 12 624 patients participated, ranging from 64 to 7565 patients per study. Two studies were unclear about the percentage of female respondents, while for the 18 other studies the majority was female (range 47–100%).

The mean age was 34.1 years (SD: 2.0) and ranged from 30 to 37 years in the 14 studies reporting on age. The time of unsuccessfully trying to have a child was on average 33.1 months (SD: 14.9; missing in eight studies). Response rate ranged from 19% to 100% (missing in seven studies). Twelve studies reported on participants’ income, nine studies included mainly participants with modal or higher than modal income, and in two studies (Palumbo *et al.*, 2011; Duthie *et al.*, 2017) at least 50% of the participants had a lower than modal income.

Except for two studies (Sills *et al.*, 2012; Huppelschoten *et al.*, 2014), the majority of participants reported having a high educational level (i.e. college or university degree), but in six studies the education level was unknown (Table 1).

### Risk of bias

**Figure 2.**
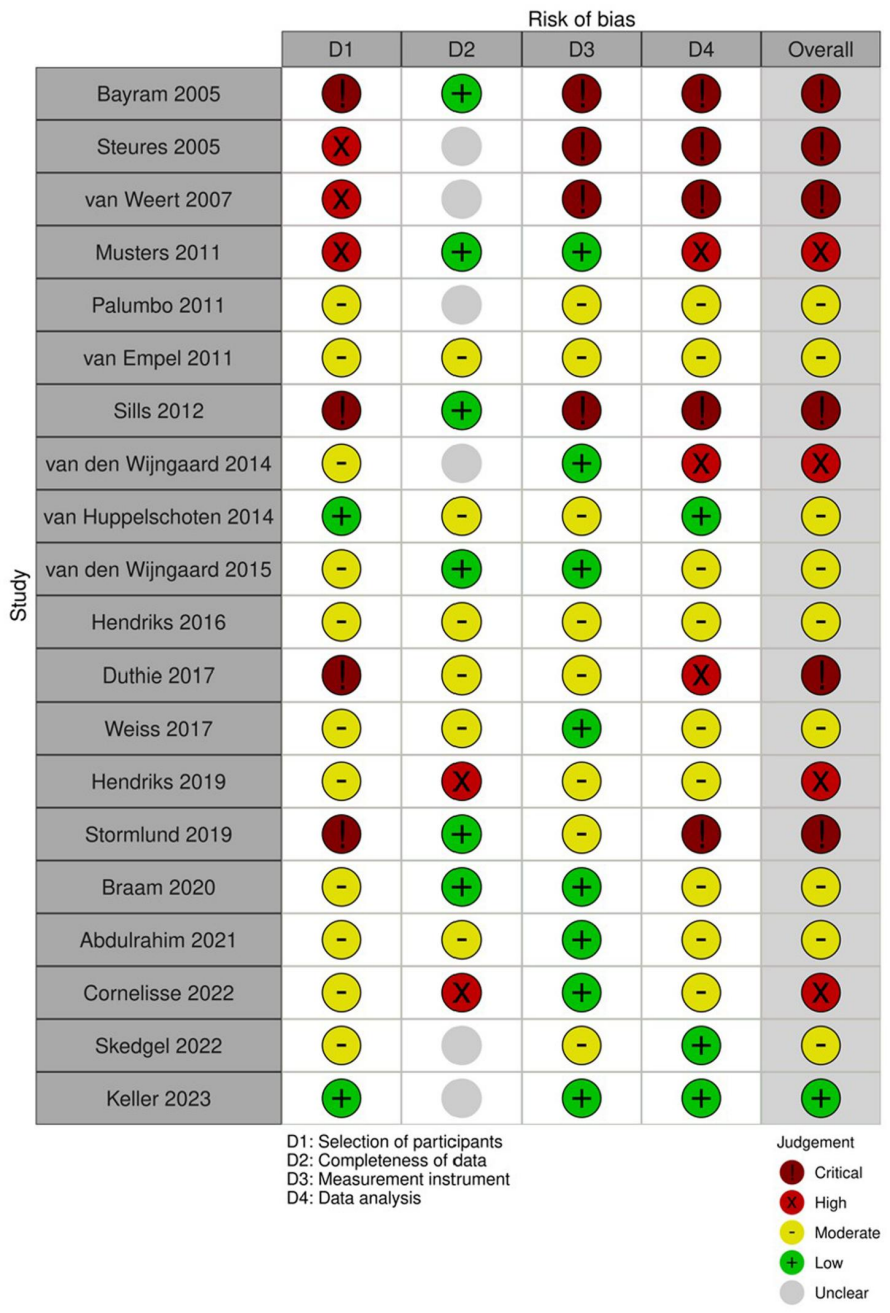
**Risk of bias assessment**.

The risk of bias rating is described in Fig. 2. All six survey/interview studies were rated as being at critical risk of bias owing to selection bias, use of unvalidated questionnaires, and not accounting for the lack of independence of the data in the analysis (multiple questions were answered by each participant) (Bayram *et al.*, 2005; Steures *et al.*, 2005; van Weert *et al.*, 2007; Sills *et al.*, 2012; Duthie *et al.*, 2017; Stormlund *et al.*, 2019). Of the 14 DCE/CA designs, nine had moderate risk of bias (Palumbo *et al.*, 2011; van Empel *et al.*, 2011; Huppelschoten *et al.*, 2014; van den Wijngaard *et al.*, 2015; Hendriks *et al.*, 2016; Weiss *et al.*, 2017; Braam *et al.*, 2020; Abdulrahim *et al.*, 2021; Skedgel *et al.*, 2022) and four had high risk of bias, for example owing to low participation rates or suboptimal statistical analysis (Musters *et al.*, 2011; van den Wijngaard *et al.*, 2014; Hendriks *et al.*, 2019; Cornelisse *et al.*, 2022). The overall rating of a study was based on the lowest rating in any of the categories. One study was scored as having a low risk of bias (Keller *et al.*, 2023) although completeness of data was unclear and selective missingness cannot be fully ruled-out; in this case, we considered the selection of participants, the instrument used and the data analysis to be the more important quality aspects.

### Attribute representation

We identified 93 attributes in the 20 included studies. The most assessed attributes were burden (23), pregnancy rate (18), safety (14), costs (12), and patient-centeredness (11). Additional attributes were genetic parenthood, natural processes (i.e. having a natural menstrual cycle and not requiring hormones), place of fertilization (i.e. insemination at hospital or intercourse at home), curing infertility (the hypothetical situation that gametes could be produced from skin cells), and singleton versus multiple pregnancy. Other attributes, only included once, were moral acceptability, technological sophistication, availability of experimental treatment (i.e. add-on treatments that are not supported by scientific evidence), and number of embryos.

#### Effectiveness

Effectiveness was reported in 17 studies (Bayram *et al.*, 2005; Steures *et al.* 2005; van Weert *et al.*, 2007; Musters *et al.*, 2011; Palumbo *et al.*, 2011; van Empel *et al.*, 2011; Huppelschoten *et al.*, 2014; van den Wijngaard *et al.*, 2014; Hendriks *et al.*, 2016, 2019; Duthie *et al.*, 2017; Weiss *et al.*, 2017; Stormlund *et al.*, 2019; Abdulrahim *et al.*, 2021; Cornelisse *et al.*, 2022; Skedgel *et al.*, 2022; Keller *et al.*, 2023). Effectiveness was operationalized by pregnancy rate or chance of pregnancy within a certain time after one treatment cycle and ranged between 2% and 50%. In one of the survey studies, patients seeking treatment to have a child were asked about ‘The importance of becoming a parent in the next year or 2’ and ‘Becoming a parent one way or another’ (Duthie *et al.*, 2017). In view of the treatment-related context of these questions, we consider this the importance of effectiveness to patients.

#### Safety

Twelve studies reported about safety (Bayram *et al.*, 2005; Steures *et al.*, 2005; van Weert *et al.*, 2007; Palumbo *et al.*, 2011; van den Wijngaard *et al.*, 2014, 2015; Hendriks *et al.*, 2016, 2019; Stormlund *et al.*, 2019; Braam *et al.*, 2020; Abdulrahim *et al.*, 2021; Skedgel *et al.*, 2022). Safety was operationalized as the chance of developing OHSS (six studies, with OHSS ranging between 1% and 10%), neonatal or child health (three studies), or maternal health or complication (three studies).

#### Costs

Costs were reported in 12 studies (Musters *et al.*, 2011; Palumbo *et al.*, 2011; Sills *et al.*, 2012; Huppelschoten *et al.*, 2014; Hendriks *et al.*, 2016, 2019; Duthie *et al.*, 2017; Weiss *et al.*, 2017; Braam *et al.*, 2020; Abdulrahim *et al.*, 2021; Skedgel *et al.*, 2022; Keller *et al.*, 2023), which differed greatly in all studies, ranging from 0 to 18 233 euros (Skedgel *et al.*, 2022). In only one study, the costs were specified as covered by national health insurance (Hendriks *et al.*, 2019). In four studies, it was specified that the costs were out of pocket for participants (Musters *et al.*, 2011; Sills *et al.*, 2012; Braam *et al.*, 2020; Keller *et al.*, 2023). In the rest of the studies, it was not specified but assumed to be out of pocket costs. In one study, cost was found to be statistically insignificant in China and highly important in the USA (Skedgel *et al.*, 2022).

#### Burden

Burden was addressed 23 times in 15 studies, and some studies reported on burden multiple times with different operationalizations (Bayram *et al.*, 2005; Musters *et al.*, 2011; Palumbo *et al.*, 2011; van Empel *et al.*, 2011; Sills *et al.*, 2012; Huppelschoten *et al.*, 2014; van den Wijngaard *et al.*, 2014, 2015; Duthie *et al.*, 2017; Weiss *et al.*, 2017; Stormlund *et al.*, 2019; Braam *et al.*, 2020; Cornelisse *et al.*, 2022; Skedgel *et al.*, 2022; Keller *et al.*, 2023). In eight studies, burden was represented by hormonal stimulation, ranging from 2 to 20 injections. Five studies reported on side effects, such as headaches, hot flushes, and insomnia. Other operationalizations of burden were risk of cycle discontinuation (two studies), travel time to the hospital (one study), number of hospital visits (three studies), importance of adherence (one study), overall treatment duration (one study), and number of treatments to achieve a 30% pregnancy rate (one study).

#### Patient-centeredness

Patient-centeredness was addressed 11 times by five studies, as some studies had multiple operationalizations of patient-centeredness (Palumbo *et al.*, 2011; van Empel *et al.*, 2011; Huppelschoten *et al.*, 2014; Skedgel *et al.*, 2022; Keller *et al.*, 2023). Four studies included the extent of patient involvement in the decision-making process (Palumbo *et al.*, 2011; Huppelschoten *et al.*, 2014; Skedgel *et al.*, 2022; Keller *et al.*, 2023). Three studies included providers’ information provision (e.g. customized versus general information), and the same studies included continuity of being treated by the same physician over time (van Empel *et al.*, 2011; Huppelschoten *et al.*, 2014; Keller *et al.*, 2023). One study also looked at physicians’ attitudes (van Empel *et al.*, 2011).

#### Genetic parenthood

Two studies examined genetic parenthood as a factor contributing to treatment decision-making (Duthie *et al.*, 2017; Hendriks *et al.*, 2019). The different situations that patients were asked to consider were absence of a genetic relation to a child in both parents, one of the parents would be a genetic parent, or both parents would be genetic parents.

### Relative importance of attributes

**Figure 3.**
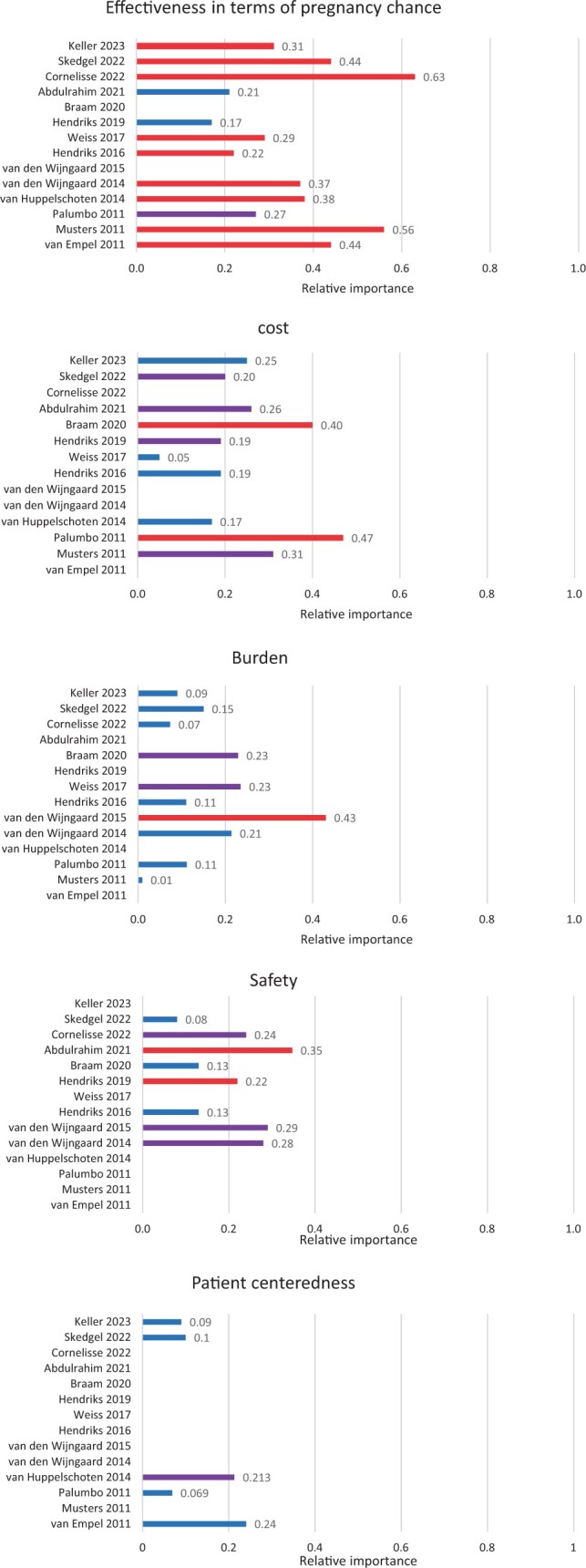
**Relative importance of effectiveness, safety, cost, burden, and patient-centeredness in decision-making related to ART.** Red = most important, purple = second most important, and blue = rest (third, fourth, fifth most important etc.).

For 14 studies, the relative importance of the attributes effectiveness, safety, burden, costs, and patient-centeredness could be determined (Musters *et al.*, 2011; Palumbo *et al.*, 2011; van Empel *et al.*, 2011; Huppelschoten *et al.*, 2014; van den Wijngaard *et al.*, 2014, 2015; Hendriks *et al.*, 2016, 2019; Weiss *et al.*, 2017; Braam *et al.*, 2020; Abdulrahim *et al.*, 2021; Cornelisse *et al.*, 2022; Skedgel *et al.*, 2022; Keller *et al.*, 2023). The relative importance of these attributes is depicted in Fig. 3. The importance of effectiveness relative to other attributes varied between 17% and 63% (median 34%) and effectiveness was rated as first or second most important factor in 10 of 12 studies (83%). In eight studies evaluating safety, the relative importance ranged from 8% to 35% (median 23%), with five studies valuing safety as first or second most important driver (63%). Regarding costs, the importance relative to the other attributes varied between 5% and 47% (median 23%), with cost being first or second most important in five of 10 studies (50%). For burden, the relative importance varied in 10 studies between 1% and 43% (median 13%) and was first/second most important in three studies (30%). Patient-centeredness had a relative importance between 7% and 24% (median 12%) and was second most important in one of the included five studies (25%).

### Trade-offs

Twelve studies provided data on trade-offs that patients would be willing to make to gain something else.

#### Burden versus pregnancy rate

It was found that women could accept additional burden in exchange for higher pregnancy rates. For instance, women would choose injections over tablets for a 6.8% increase in pregnancy rates; as well as two hospital visits instead of none in exchange for an 8.9% increased pregnancy rate. (Weiss *et al.*, 2017). Women were willing to accept a 5.2% decrease of pregnancy rate for 75 minutes less travel time (van Empel *et al.*, 2011). Women were less willing to trade-off pregnancy rates for less burdening side-effects, importance of adherence, and duration of treatment. For less side effects, women were willing to accept a 0.8% decrease in pregnancy rate, for less importance of adherence this was 0.4%, and for a shorter duration of treatment, this was 0.3% (van den Wijngaard *et al.*, 2014).

#### Safety

Participants were willing to accept a 1.3% reduction in the live birth rate in exchange for a 1% reduction in the risk of neonatal complications (Abdulrahim *et al.*, 2021). Enduring more injections would be traded for a 6.2% decrease in risk of cycle cancellation or a 4.5% decrease in chance of OHSS (van den Wijngaard *et al.*, 2015). Participants would also trade-off a 0.9% lower pregnancy rate for an OHSS risk reduction of 1.6% (van den Wijngaard *et al.*, 2014). Women were willing to accept a 5.0% higher risk of cycle cancellation if the OHSS rate decreased by 2% (Braam *et al.*, 2020). In addition, women accepted one extra IVF cycle for a reduction of 3.9% in OHSS risk (Braam *et al.*, 2020).

#### Patient-centeredness

Patients would trade-off mean ongoing pregnancy rate for being treated with more patient-centeredness. One study found patients were willing to sacrifice 9.8% of pregnancy rate if being treated by a friendly, interested physician, or sacrifice 9.6% of pregnancy rate if getting clear and tailored information instead of contradictory information (van Empel *et al.*, 2011). Continuity of physicians was worth a 4.0% decrease of pregnancy rate (van Empel *et al.*, 2011).

#### Genetic parenthood

Although genetic parenthood did not influence decision-making statistically, one study stated that: ‘Patients would accept non-genetic parenthood over genetic parenthood in return for a pregnancy rate increase of 18%, a child health risk reduction of 3.6%, a cost reduction of €3500, an OHSS risk reduction of 4.6%, or a maternal cancer risk reduction of 2.7%’ (Hendriks *et al.*, 2019).

#### Willingness to pay

More than half of the participants of one study would not pay extra for gains in comfort of drug administration or local tolerance of injections (Palumbo *et al.*, 2011). In contrast, one study found patients were willing to pay more to avoid moderate side-effects €1303–€1786) and significant side effects (€2133–€2923), but the nature of these treatment-related side effects was not specified (Keller *et al.*, 2023). Patients were also willing to pay €1000 for a decrease in OHSS rate of 5.4% (Braam *et al.*, 2020).

In addition, patients were willing to pay a median of €463 for improved patient-centeredness (e.g. clearer information and more patient involvement; Huppelschoten *et al.*, 2014), with higher income and age associated with greater willingness to pay. In the study of Skedgel *et al.* (2022), patients from Nordic countries and the USA would pay up to €3000 for ‘full’ shared decision-making.

#### Willingness to pay for higher pregnancy rate

The willingness to pay for improved pregnancy rates was evaluated by six studies and all found that patients were prepared to pay for more effectiveness. In one study, patients would pay €107 for 1% higher pregnancy rates (Huppelschoten *et al.*, 2014), which is consistent with another study where 36% of participants were willing to pay €101–€300 for a 1–2% increase (Weiss *et al.*, 2017). This is in the same range as €1000 in exchange for 5–14% increased pregnancy rates (Musters *et al.*, 2011) or an additional €500 in exchange for an 8.9% increase in pregnancy rate (Palumbo *et al.*, 2011). An Australian study (Keller *et al.*, 2023) found patients were willing to pay €232–€317 for 1% increase in pregnancy rate, which is considerably more than in the European countries. Overall, the willingness to pay was dependent on patients’ income. Likewise, patients were willing to pay more for an increased likelihood of a live birth than for an improvement in any other attribute (Skedgel *et al.*, 2022). Thereby, it was noted that ‘*Nordic countries, Spain and the UK were willing to pay between €3000 and €3500 for a 15% absolute improvement in effectiveness, whilst respondents from the USA were willing to pay more than €6000*’ (Skedgel *et al.*, 2022).

**Figure 4.**
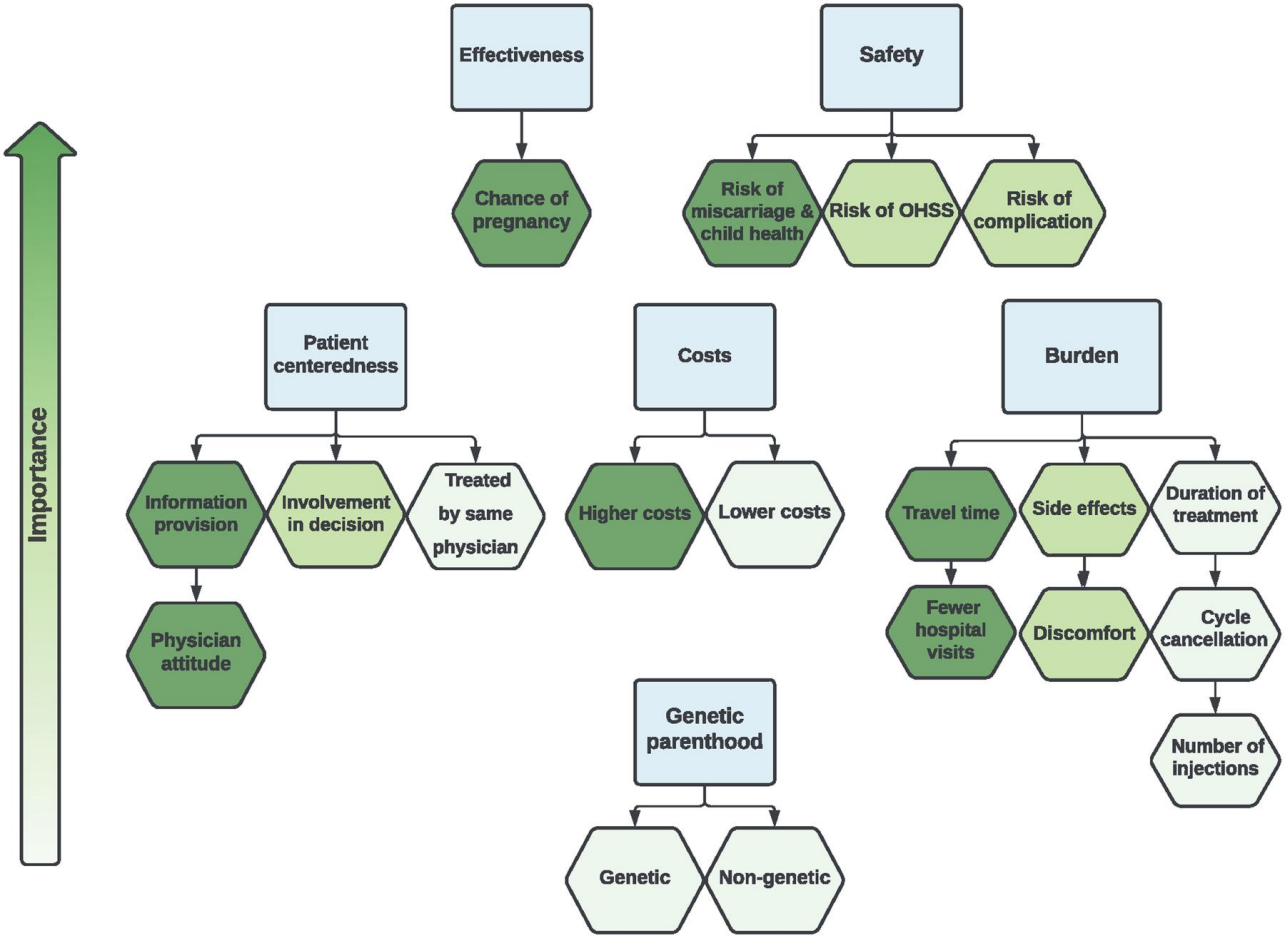
**Visual representation of relative importance of attributes.** Darker color represents higher priority of attributes.

Based on the relative importance of attributes and trade-offs, a schematic diagram was developed (Fig. 4).

### Results of survey and interview studies

In five preference studies, effectiveness was seen as most important, but the child’s health was also regarded as very important in these studies (Bayram *et al.*, 2005; Steures *et al.*, 2005; van Weert *et al.*, 2007; Duthie *et al.*, 2017; Stormlund *et al.*, 2019). Stormlund *et al.* (2019) found that women were even willing to accept risks to their own health in order to achieve a pregnancy. However, Hendriks *et al.* (2016) found that the safety of the child was slightly more important than pregnancy rates. In the study of Sills *et al.* (2012), effectiveness was not studied, but cost and burden were of equal importance. When costs were low (i.e. €90) most patients would pay extra for fewer injections, but not when costs increased.

Besides effectiveness, burden also played an important role in the study of Bayram *et al.* (2005), with some patients never opting for the offered treatment because they did not want to undergo surgery or inject hormones. In addition, genetic parenthood and costs were seen as a moderate priority (Duthie *et al.*, 2017). As a hypothetical option, curing infertility was the third most important factor in one study (Hendriks *et al.*, 2019). Costs, burden, natural process, and technological sophistication were of about equal importance in the same study.

Conceiving at home and moral acceptability of ART were found to be less important than the attributes mentioned earlier (Hendriks *et al.*, 2016). A longer waiting time for embryo transfer and a more natural process without hormones were also less important in the decision-making process (Stormlund *et al.*, 2019). Avoiding side effects was not as important to women as effectiveness or genetic parenthood in one study (Duthie *et al.*, 2017). Risk of twin pregnancies was not important (Steures *et al.*, 2005; van Weert *et al.*, 2007).

## Discussion

With this systematic review, we aimed to determine which attributes are valued most by ART patients with an unfulfilled wish to have a child. We identified 93 attributes in 20 studies that were classified as effectiveness, safety, burden, cost, patient-centeredness, genetic parenthood, and others. The most assessed attributes were burden, effectiveness (pregnancy rate), safety, costs, and patient-centeredness. The relative importance of attributes was established in 14 studies. We found that effectiveness was rated as the first or second most important factor in 83%, safety in 63%, cost in 50%, burden in 30%, and patient-centeredness in 25% of studies. This was in line with the six survey and structured interview studies that also found attributes related to effectiveness, safety, cost, and burden having most impact on decision-making.

A strength of this study is that we calculated the relative importance of the different attributes, thus enabling us to make more objective statements on the order of importance of the attributes. Although the studies included in this review were conducted in many different countries, patient preferences were very similar. This overlap in findings suggests that patient preference studies regarding ART treatments may be generalizable across different populations.

Although we limited our review to particular attributes of ART, by design, the included studies differed in assessed attributes and methodology. Effectiveness was included in almost every study, while genetic parenthood and other attributes, such as curing infertility, place of fertilization, chance of miscarriage, and importance of adherence, were only included in one or two studies; owing to the lack of representation in the studies, we were unable to assess their influence and therefore do not know whether these aspects are of importance to patients.

A large proportion of the included studies was performed in Europe, particularly in the Netherlands, and, although comparable choices were made across studies and suggest generalizability, we did find some notable differences between countries both within and between the studies. Within the largest study (Skedgel *et al.*, 2022) among patients from the USA, China, and Europe, it was found that patient-centeredness and costs were not considered important in China, while particularly costs were almost as important as effectiveness in the USA. The authors suggest that this was related to differences in the health and reimbursement system. Similar to the USA, the Australian study (Keller *et al.*, 2023) observed that patients were prepared to pay more for treatment success than in Europe, possibly due to less of the costs being reimbursed, resulting in significant out-of-pocket costs.

It is advisable to ascertain patients’ preferences before recommending certain treatments. Preference measurement approaches, particularly DCEs, have been shown to be effective instruments to understand decision-making in patients, as well as in providers and administrators (Salloum *et al.*, 2017). Furthermore, although the choices that are made in the studies may differ from those made in real life (Hendriks *et al.*, 2019), a previous study found that DCEs have moderate accuracy when predicting health-related choices (Quaife *et al.*, 2018). In preference research, the non-random selection of participants and selection bias owing to non-response jeopardized the external validity. Such non-response tends not to be random, and most studies include, for example, highly educated patients earning (more than) modal income. Strategies need to be implemented to ensure that the participants included are representative of the target population.

### Findings in context

Pregnancy rate was often only slightly more important than safety and never more important than child health risks. Two studies did not value effectiveness (pregnancy rate) as the most important attribute (Hendriks *et al.*, 2019; Abdulrahim *et al.*, 2021). In both of these studies, half of study participants were male partners, and both included neonatal complication as a safety attribute, which other DCEs did not include. In both studies, preventing neonatal complication was considered most important, particularly by the (male) partner.

In our review, burden was found to be less important when having to choose between different attributes, which is consistent with the study by Dancet *et al.*, (2011) that found ‘physical burden’ to be one of the least important aspects. Likewise, a review indicated that physical burden of treatment was of minimal importance during first-order treatments, while psychological burden was a frequently named reason for patients to drop-out of the fertility program (Gameiro *et al.*, 2012). This may imply that women are willing to endure quite some burden to have a child before starting ART, but that the reality of going through such treatments can be harder than expected (Cousineau and Domar, 2007; Copp *et al.*, 2020). Unfortunately, psychological burden was not included as an attribute in the studies included in our review and may be considered in future preference studies. Much is still unknown about how psychological constructs relate to preference formation or differences in preferences between participants in general (Russo *et al.*, 2019). However, in a recent study of patients experiencing unsuccessful IVF/ICSI cycles, 93.3% expressed a desire for psychosocial care, particularly when faced with a poor prognosis. Participants emphasized the need for personalized information on treatment options, outcomes, and comprehensive psychosocial support, as well as addressing coping strategies to process loss and sustain hope toward the future (Sousa-Leite *et al.*, 2023).

Our findings on patient-centeredness are largely consistent with the literature. In line with a previous review, we found information provision to be an important aspect of patient-centeredness, together with time for discussions, personalized care, and friendly and interested providers (Dancet *et al.*, 2010). Another study looked particularly at the role of the doctor and partner in decision-making in women with an unfulfilled wish to have a child. In this study, patient involvement in the decision-making process was valued as important by 92% of the patients (Chan *et al.*, 2019). Patient involvement remains an attribute that needs further investigation.

Studies report different views on the importance of genetic parenthood. Two studies reported the importance of genetic parenthood for patients (Hendriks *et al.*, 2017; Duthie *et al.*, 2017), while another study reported that genetic parenthood did not influence decision-making (Hendriks *et al.*, 2019). This could be partially explained by differences in study participants, where those who have tried to have a child for longer (Hendriks *et al.*, 2019) may be more willing to accept non-genetic parenthood than patients who are in the earlier stages of infertility treatment (Duthie *et al.*, 2017). Most couples in a study conducted in Iran had a preference for biological children rather than children conceived with donor gametes or adopted children, despite repeated treatment failure, low success rate, and high costs of infertility treatment (Behjati Ardakani *et al.*, 2022). Cultural differences or sexual orientation may further explain the different levels of importance placed on genetic parenthood.

### Implications for practice

The findings from this review can be used to inform patient-centered care guidelines. One of the key aspects of clinical practice guidelines is to provide information about the benefits and harms of specific interventions or treatments, to help healthcare providers and patients to make informed decisions that consider individual patients’ needs and preferences (Huppelschoten *et al.*, 2013). Acknowledging the importance of information about all aspects of different treatment options can help to promote transparency in medical decision-making (Gärtner *et al.* 2019).

### Implications for further research

To date, mental and psychological factors (e.g. burden) of fertility treatments remain understudied and could enrich research and contribute to better patient experiences and care in the future. Qualitative studies will be needed to find understudied attributes that are potentially relevant to patients. To study the importance of attributes and trade-offs, the preferred methodology is the DCE as this likely provides the most objective information. New DCEs may consider assessing an opt-out option as this is more representative of reality, rather than forcing patients to make a choice between treatment options when they might be in doubt.

The identification of driving factors in treatment decision-making for ART may stimulate further research. It would be of value to study whether the importance of driving factors shifts over time or over treatment trajectories. It is also unknown whether these factors influence risk of drop-out.

## Conclusion

We found effectiveness of treatment, followed by safety, costs, and burden as the most important attributes determining patient preferences for ART. Patient-centeredness should also be considered in clinical practice and research, although its impact on decision-making is still unclear. Patients were prepared to trade-off some effectiveness for more safety, less burden, more patient-centeredness and were prepared to pay for more effectiveness.

The results of this review can be used in future preference studies, while they can also help healthcare professionals in guiding patients’ decision-making and enable a more patient-centered approach.

## Supplementary Material

dmae001_Supplementary_Data

## Data Availability

The data underlying this article are available in the article and in its Supplementary Material.
