# Supplementary material for: Driving factors in treatment decision-making of patients seeking medical assistance for infertility: a systematic review
Source: Hum Reprod Update. 2024 Feb 1;30(3):341–54. doi: 10.1093/humupd/dmae001 (PMC11063545; doi:10.1093/humupd/dmae001)
Supplement: dmae001_Supplementary_Data [file dmae001_supplementary_data.docx]

**Supplementary Data File S1**

**Searches**

***EMBASE:***

*patient preference.mp. or exp patient preference/ AND exp infertility/ or exp infertility therapy/ or exp ovulation induction/or exp intrauterine insemination/or exp fertilization in vitro/or exp intracytoplasmic sperm injection/ or exp cryopreservation/ or exp embryo transfer/*

***Psycinfo:***

*exp Preferences/ or patient preference.mp AND exp Infertility/or exp Reproductive Technology/ or ovulation induction.mp. /or intra uterine insemination.mp. /or in vitro fertilization.mp.*

***Pubmed:***

*(patient preference[MeSH Terms] OR (patient preference[All Fields]) AND ((infertile[All Fields] OR infertility[MeSH Terms] OR infertility[All Fields] OR (ovulation induction[MeSH Terms]) OR (intra-uterine insemination[All Fields])) OR insemination[MeSH Terms] OR IVF[All Fields] OR ART[All Fields] OR (sperm injections, intracytoplasmic[MeSH Terms]) OR ICSI[All Fields] OR (embryo transfer[MeSH Terms]) OR (embryo transfer[All Fields])*

***CINAHL:***

*(infertility and discrete choice experiment OR DCE) OR (preference and infertility(major heading))*
